# The Evolution of Sexual Fluids in Gymnosperms From Pollination Drops to Nectar

**DOI:** 10.3389/fpls.2018.01844

**Published:** 2018-12-18

**Authors:** Patrick von Aderkas, Natalie A. Prior, Stefan A. Little

**Affiliations:** Centre for Forest Biology, Department of Biology, University of Victoria, Victoria, BC, Canada

**Keywords:** gymnosperm, nectar, nucellus, ovular secretion, pollination drops, pollen, ovule

## Abstract

A current synthesis of data from modern and fossil plants paints a new picture of sexual fluids, including nectar, as a foundational component of gymnosperm reproductive evolution. We review the morpho-anatomical adaptations, their accompanying secretions, and the functional compounds involved. We discuss two types of secretions: (1) those involved in fertilization fluids produced by gametophytes and archegonia of zooidogamous gymnosperms, i.e., *Ginkgo* and cycads, and (2) those involved in pollen capture mechanisms (PCMs), i.e., pollination drops. Fertilization fluids provide both liquid in which sperm swim, as well as chemotactic signals that direct sperm to the egg. Such fertilization fluids were probably found among many extinct plants such as ancient cycads and others with swimming sperm, but were subsequently lost upon the evolution of siphonogamy (direct delivery of sperm to the egg by pollen tubes), as found in modern gnetophytes, conifers, and Pinaceae. Pollination drops are discussed in terms of three major types of PCMs and the unique combinations of morphological and biochemical adaptations that define each. These include their amino acids, sugars, calcium, phosphate and proteins. The evolution of PCMs is also discussed with reference to fossil taxa. The plesiomorphic state of extant gymnosperms is a sugar-containing pollination drop functioning as a pollen capture surface, and an *in ovulo* pollen germination medium. Additionally, these drops are involved in ovule defense, and provide nectar for pollinators. Pollination drops in anemophilous groups have low sugar concentrations that are too low to provide insects with a reward. Instead, they appear to be optimized for defense and microgametophyte development. In insect-pollinated modern Gnetales a variety of tissues produce sexual fluids that bear the biochemical signature of nectar. Complete absence of fluid secretions is restricted to a few, poorly studied modern conifers, and is presumably derived. Aspects of pollination drop dynamics, e.g., regulation of secretion and retraction, are reviewed. Lastly, we discuss pollination drops’ control of pollen germination. Large gaps in our current knowledge include the composition of fertilization fluids, the pollination drops of Podocarpaceae, and the overall hydrodynamics of sexual fluids in general.

## Introduction

Fluids play major roles during reproduction of gymnosperms. Ovule-derived fluids are almost universally found in pollen capture mechanisms (PCMs). In addition, early diverging gymnosperms are dependent on fluids for fertilization, not just for pollen capture. Before looking at the nature and complexity of these aqueous fluids it is necessary to introduce some of the aspects of reproduction that are unique to gymnosperms, beginning with pollination and then proceeding to fertilization.

**FIGURE 1:**
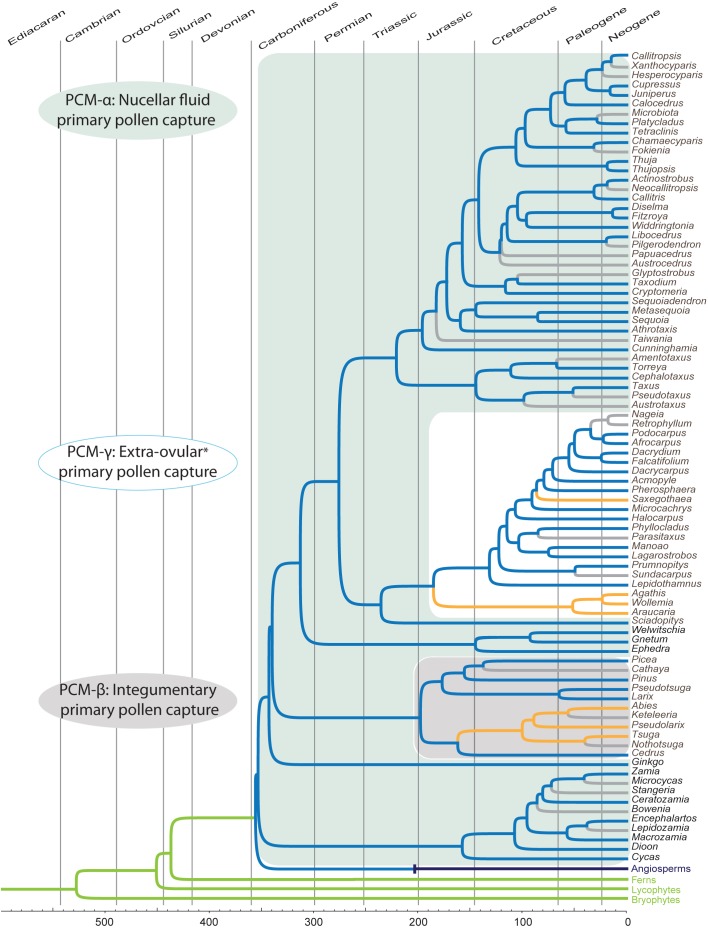
Chronogram of the extant genera of gymnosperms based on Lu et al. (2014), and Clarke et al. (2011) for relationships, and divergence times, of angiosperms and free-sporing plants. Blue branches represent presence of pollination drops *sensu lato* (i.e., where ovular secretions from the nucellus appear between pollen capture and fertilization). Gray branches represent missing data. Yellow branches represent well-studied taxa that have been reported to lack nucellar ovular fluids in their pollination (pollination drops, *sensu lato*). Green branches represent free-sporing sex, whether homo- or heterosporous. Purple branch for angiosperms represents flower-based sex; the origin is based on one of the divergence times from Clarke et al. (2011). Light blue enclosing rectangle represent the case of the most common pollen capture mechanism among extant taxa, PCM α: nucellar fluid performing the functions of: (i) capture of non-saccate pollen, (ii) delivery of pollen into the ovule interior, (iii) germination medium of pollen, and (iv) ovule defense. Gray rectangle represents the shift to primary pollen capture by integuments in Pinaceae, PCM β. White rectangle represents the shift to various ovular, and extra-ovular primary capture mechanisms (PCM γ) in Podocarpaceae *sensu lato* and Araucariacae. Note that *Saxegothaea*, and Araucariaceae lack drops. Extinct fossil seed plants not shown; the earliest plants with seed-like structures appear in the Upper Devonian. Data for drop presence/absence from: Norén (1908), Tison (1911), Saxton (1913a,b), Doyle and Saxton (1932), Brough and Taylor (1940), Doyle (1945), Dogra (1964), Tang (1987, 1993), Takaso (1990), Tomlinson (1991, 1992, 1994), Tomlinson et al. (1991, 1997), Carafa et al. (1992), Takaso and Tomlinson (1992), Kato et al. (1995), Molloy (1995), Owens et al. (1995), Takaso and Owens (1996a, 2008), Möller et al. (2000), Mill et al. (2001), Zhuowen (2004), and Li and Huang (2006).

**FIGURE 2:**
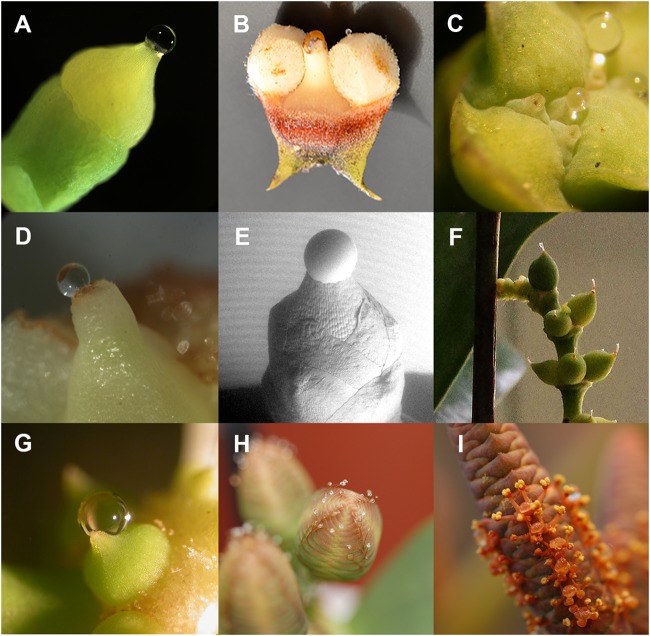
Pollination drops **(A)** *Ginkgo biloba*, **(B)** *Ceratozamia hildae*, **(C)** *Tetraclinis articulata*, **(D)** *Pseudotsuga menziesii* (post-pollination prefertilization drop), **(E)** *Taxus* x *media* (scanning electron micrograph by A. Lunny), **(F)** *Gnetum gnemon* female, **(G)** *G. gnemon* male, **(H)** *Welwitschia mirabilis* female, and **(I)** *W. mirabilis* male.

A critical feature of gymnosperm pollination is that in almost all species the primary capture surface for pollen is an ovular secretion (Williams, 2009). Generally, this is called a pollination drop (Singh, 1978). Some angiosperm ovules are able to secrete fluids that influence pollen tube behavior (Franssen-Verheijen and Willemse, 1993). Ovules secrete a fluid that fills the micropyles, which attracts pollen tubes into the ovule where the pollen tube breaches the relatively thin nucellus before depositing male gametes into the embryo sac. Angiosperm ovular secretions are relatively unknown compared to pollination drops of gymnosperms. Pollination drops are a common part of extant gymnosperm pollination biology (Figure 1), and are found in all modern clades: *Ginkgo* (Figure 2A), cycads (Figure 2B), conifers (Figures 2C–E), and Gnetales (Figures 2F–I). These liquid-based interactions between ovule and pollen are likely to be of ancient origin. Pollination drops provide a number of conserved functions that are essential components of mechanisms involved in pollen capture, delivery, and germination. Pollination drops also provide ovule defense against microbes during reproduction (Little et al., 2014).

A distinctive aspect of some gymnosperms, and one that we will develop further in this review, is that ovules are able to secrete pollination drops that also double as attractants to pollinators. Gymnosperms that are insect-pollinated fall into two types: those that are ambophilous, i.e., the plants receive pollen by insects and wind (Meeuse et al., 1990; Kono and Tobe, 2007; Gong et al., 2016), and those that have obligate pollination mutualisms with insects, e.g., some cycads (Mound and Terry, 2001) and gnetophytes (Tang, 1987; Kato and Inoue, 1994). Although pollination drops mediate pollen capture in both types, among those that have obligate pollination mutualisms is a group of gnetalean species that reward pollinators with nectar produced by ovules (Kato et al., 1995). The evolution of nectar from pollination drops is unique to gymnosperms and will be discussed in greater depth.

In addition to fluid produced during pollination, ovules may also produce fluids during fertilization. Fertilization fluids are common to archegoniate plants, e.g., mosses, ferns and gymnosperms. These plants reproduce by means of eggs that are found inside the archegonium, the female sex organ whose presence sets gymnosperms apart from angiosperms. The structure of an archegonium is simple. A well differentiated, relatively large egg is found at the base. Above the egg, in the case of gymnosperms, is one cell; in the case of mosses and ferns, there are two cells. These cells are surrounded by neck cells, which are an adaptation to fluid-based reproduction. Upon wetting, neck cells part to allow the contents of the cells above the egg to be released. Sperm swim down this now fluid-filled passage to the egg where fertilization takes place. Whereas ferns and mosses need free water to reproduce, gymnosperms, such as *Ginkgo* and cycads, produce their own fluid. In short, reproduction with archegonia requires an aqueous medium for sperm delivery. Eventually, gymnosperm groups evolved for which this fluid requirement was bypassed.

**FIGURE 3:**
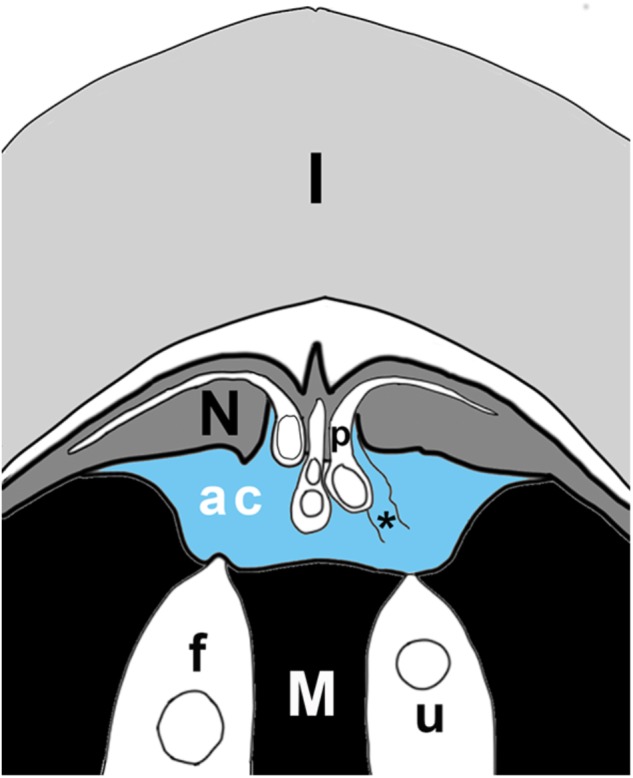
Schematic of ovule tip at time of fertilization, showing layers of integument (I), nucellus (N), megagametophyte (M), with two archegonia (in white), of which one is fertilized (f), the other unfertilized (u). Pollen tubes (p) have grown into the nucellus; the sulcus end of the tube hangs over the archegonial chamber (ac). The archegonial chamber may be filled with fluid (blue) that originates either from ruptured pollen tubes (asterisk), from cells of the megagametophyte that line the chamber, and/or from archegonia. Some published accounts state that fluids from megagametophytes may be sufficient to fill the chamber (blue), or may be much less abundant, having only the fluids of a few ruptured pollen tubes mixed with secretions from archegonia. In the plant, the orientation of the ovule is reversed, with the megagametophyte at the top. Figure is based on Chamberlain’s (1910) illustration of *Dioon edule* ovule.

Water is the most abundant molecule in a sexual fluid, and is important to both fertilization and to pollination in gymnosperms. However, this water is mainly a solvent for compounds that influence microgametophyte-ovule interactions. As mentioned above, early diverging embryophytes, such as mosses and ferns, are entirely dependent on water for reproduction. Since their sperm need water in which to swim it would at first appear that they do not contribute sexual fluids to this process. However, mosses and ferns release a fluid from their archegonia that is developmentally timed to assist in fertilization. When an egg ripens, the other cells within the archegonium and above the egg, i.e., neck canal cell and ventral canal cell, break down and die. The contents of these dead cells are released into the surrounding free water after the necks have separated. Contents of the dead cells further improve the chances of fertilization by creating the chemical gradients that set up sperm chemotaxis. Moss sperm were thought to be attracted to archegonia by a gradient of released sucrose (Ziegler et al., 1988). Recently, Ortiz-Ramirez et al. (2017) found that sperm chemotaxis in the moss *Physcomitrella patens* depended upon sperm ionotropic glutamate receptors. However, the specific ligand released by the archegonia that triggers this chemotactic response by the sperm remains unknown. Archegonial secretion of chemoattractants also occurs in some gymnosperms (Figure 3). Gymnosperms such as cycads release fluids during fertilization (Chamberlain, 1935). One such fluid is that released by megagametophyte tissues surrounding their archegonia (Takaso et al., 2013). This fluid fills the specialized fertilization chamber in which the archegonia are found (Figure 3). Once this chamber is filled, sperm are released from the pollen tubes and the archegonial neck cells divide forming a four-celled neck apparatus, centrally open to the egg. Archegonia release copious amounts of a white-colored substance that appears to play a role in chemotaxis (Takaso et al., 2013). Swimming sperm delivery via a microgametophyte with haustorial pollen tubes is known as zooidogamy and is characteristic of earlier diverging gymnosperms (Williams, 2009), such as *Ginkgo* and cycads. More derived gymnosperms produce gametes that are delivered by a linear pollen tube, but these gametes lack flagellae and, therefore, cannot swim. Instead, pollen tubes deliver the male gamete directly into the egg. This is called siphonogamy and occurs in all extant lineages of conifers and Gnetales. However, they sometimes still have archegonial chambers, albeit small ones, such as those found in *Picea* (Runions and Owens, 1999). The neck cells and neighboring cells surrounding the archegonium secrete lipid into the chamber space. These lipids are thought to be essential in signaling and directing pollen tubes to their destination (Runions and Owens, 1999).

It is the purpose of this review to trace the evolution of sexual fluids in gymnosperms, to describe the aspects of their biochemistry that we currently understand, as well as to suggest future directions of investigation. This review also has a particular emphasis, which is to trace the unique origins of gymnosperm nectar.

## Modern Gymnosperms

Pollination drops are widespread among modern gymnosperms, archegonial chamber fluids less so. Pollination drops are produced by the ovule’s diploid nucellus, whereas archegonial fertilization fluids are produced by the ovule’s haploid gametophytes. We will discuss archegonial chamber fluids first. Although their role in sexual reproduction is clear, details of their composition are the most poorly understood of all of the gymnosperm sexual fluids.

### Archegonial Chamber Fluid – Function and Composition

This fluid is mainly restricted to cycads and *Ginkgo*, the extant zooidogamous gymnosperms. Since the process of secretion takes place inside the ovule it is difficult to observe. Accounts of events are mostly of a descriptive, rather than experimental nature. For thorough historical discussions, see Hori and Miyamura (1997) and Norstog and Nicholls (1997). There are conflicting views as to the origins of the fluid(s). Three origins have been proposed. The first of these is the pollen tube. In *Dioon edule*, as pollen tubes rupture during sperm release, they release a fluid that is of sufficient volume (Figure 3) to provide a thin film in which the sperm are able to swim (Chamberlain, 1910). If pollen tubes are numerous, they may even release enough fluid to fill the entire archegonial chamber (Brough and Taylor, 1940). A second source is the megagametophyte. In *Cycas revoluta*, fluids are released from megagametophyte cells lining the archegonial chamber (Figure 3). Cells at the rim of the depression secrete first, followed by cells at the base (Takaso et al., 2013). A third source of fluid is from individual archegonia. In *Ginkgo biloba*, archegonial neck cells release fluid (Wang et al., 2014). Combinations of fluids are also possible, e.g., archegonial and pollen tube fluids (Chamberlain, 1910).

Some experimental work provides evidence for the functions of these fluids. In a study of pollen tubes in different conditions, Takaso et al. (2013) found that turgid pollen tubes had to be in contact with archegonial chamber fluid for a number of hours before they were able to discharge their sperm. The possibility that there may be a degree of molecular interaction between secreted pollen proteins and ovules that could be considered as a form of a recognition system was first put forward by Pettitt (1977) in his study of cycads. Pettitt’s inferences were based on protein gels run from extracted whole ovules, rather than isolated fluids. He considered the context of these fluids, recognizing that the archegonial chamber fluids occur at the interface between the haploid megagametophytes and the surrounding diploid sporophytic ovule tissue. These genetically different tissues are separated from one another by a megaspore wall, which is a thick, complex structure composed of glycoproteins, cellulose, hemicellulose, and sporopollenin. The sporophyte-gametophyte *Bauplan* of the ovule imposes communication constraints (Williams, 2009). The physiological isolation that this wall imposes prevents interactions between the gametophyte and the sporophyte (Pettitt, 1979). Unfortunately, no molecular studies of protein interactions during reproduction have been carried out since these papers appeared. Even an initial analysis of archegonial chamber fluid composition has yet to be carried out. Detailed proteomic and metabolomic analysis of these fluids would add significant information to our understanding of the evolution of sperm-ovule interactions, from sperm discharge and chemotaxis through to ovule defense.

Archegonial secretions and neck canal secretions have been mainly studied by transmission electron microscopy. In both cycads with their large archegonial chambers (Takaso et al., 2013) and pinaceous conifers, e.g., Douglas-fir (Takaso and Owens, 1994) and spruce (Runions and Owens, 1999) with their small archegonial chambers, there is evidence of lipid secretion. These lipids have never been isolated and analyzed. Although collection of archegonial secretions may appear to pose sampling difficulties, with today’s ultrasensitive mass spectrometers, even small samples are likely to provide results.

### Pollination Drops and Related Secretions and Their Role in Pollen Capture Mechanisms

Among modern gymnosperm taxa, species have various pollination syndromes, i.e., whether they are wind pollinated and/or insect pollinated, and more specifically according to their mechanisms for collecting pollen. These mechanisms make use of secretions, i.e., lipid microdrops and/or nucellar fluids, or similar secretions. However, in a small number of species there are mechanisms that do not use secretions as far as we know (Gelbart and von Aderkas, 2002). Such mechanisms are restricted to the conifer family, Araucariaceae (Eames, 1913; Haines et al., 1984; Owens et al., 1995), and some genera of Pinaceae (Doyle and O’Leary, 1935b; Doyle and Kane, 1943), and Podocarpaceae (Figure 1; Doyle and O’Leary, 1935a; Tomlinson, 2012; for a detailed review, also see Little et al., 2014). We will only touch on these mechanisms throughout; this review focuses on cases of sexual secretions and possible nectars.

**FIGURE 4:**
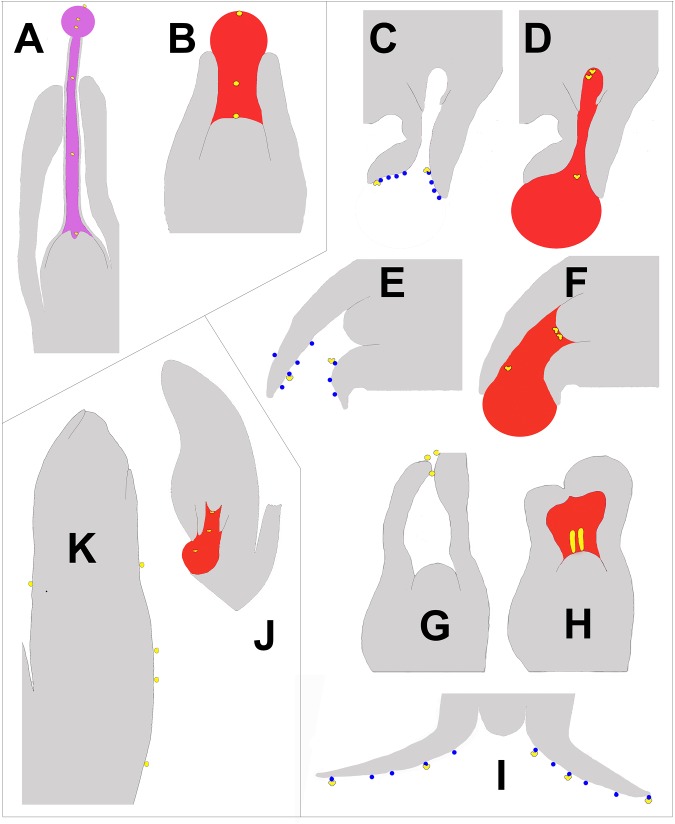
Schematic of longitudinal sections of portions of ovules at time of pollination illustrating the three types of pollen capture arranged clockwise. Nectar is pink, pollination drops are red, and lipid microdrops are blue. Pollen is yellow and is either round or saccate, depending on the mechanism. **(A)** *E. foeminea –* PCM α (after Rydin et al., 2010). The lowest pollen grain can be seen entering a depression in the nucellus known as the pollen chamber, which is formed by PCD. **(B)** *Taxus canadensis –* PCM α (after Dupler, 1920), **(C,D)** *Picea sitchensis –* PCM β (after Owens and Blake, 1985). The two uppermost pollen grains can be seen floating into a pollen chamber. **(E,F)** *Cedrus deodora –* PCM β (after Takaso and Owens, 1995a,b). **(G,H)** *Larix decidua –* PCM β (modified from Doyle, 1945). **(I)** *Abies amabilis –* PCM β (modified after Chandler and Owens, 2004). **(J)** *Podocarpus –* PCM γ (after Doyle, 1945). **(K)**. *Agathis australis –* PCM γ (after Owens et al., 1995).

Pollen capture mechanisms have been classified in several ways in the past. Traits such as pollen morphology, ovule orientation, and timing (and/or the lack) of ovular secretions have been used (Little et al., 2014). Here, we divide the modern variation known into three categories based on their primary pollen capture surface (Figure 1). The most widespread and ancient is PCM α (Figure 1; blue enclosed area), in which a nucellus-based ovular fluid extrudes from the ovule to act as the primary capture surface for pollen. This liquid surface is the first contact that pollen has with the ovule. The second major category, PCM β, has primary pollen capture by integuments, as found in Pinaceae (Doyle and O’Leary, 1935a; Doyle and Kane, 1943). Some species have a drop that appears later and brings pollen into the ovule. The third category, PCM- γ, represents pollen capture by an extra-ovular surface, typically by cone surfaces adjacent to the ovules, as observed in some Podocarpaceae (Figure 4J; Doyle and O’Leary, 1935b; Tomlinson, 1994).

Nectar is known only from the most prevalent type, PCM α. This nucellus-based ovular fluid also performs a myriad of functions, which include primary pollen capture, pollen delivery into the ovule, pollen germination, and defense of the ovule against pathogens.

We present a synthesis using the well-sampled genera-level phylogeny of Lu et al. (2014), and rely on their divergence time estimates to illustrate the origins and evolution of sexual fluids in extant gymnosperms (Figure 1). Additional divergence times and phylogenetic relationships come from Clarke et al. (2011). The presence of nucellar secretions at the pre-fertilization stage of the seed, i.e., pollination drop *sensu lato*, has been recorded and mapped on the chronogram (blue branches). The presence of the drop among modern gymnosperm clades is widespread (Figure 1). Regardless of possible future alternative phylogenetic hypotheses, it seems very likely that the foundational nature of ovular fluids will remain a robust inference. This is due, in part to the prevalence of sexual fluids among the majority of modern gymnosperm groups and thus the ancestral condition of having a pollination drop would be similar among all major lineages given alternative topologies (i.e., Zhong et al., 2011; Xi et al., 2013; Ruhfel et al., 2014; Wickett et al., 2014).

#### Pollen Capture Mechanism- α -Wind and/or Animal Pollination

In the most common PCM α (Figure 1), the nucellus secretes a pollination drop that first fills the micropyle, the cavity at the apex of the ovule (Figures 4A,B). As secretion continues, a fluid balloons outward from the opening of the micropyle in a spherical drop. In these ovules, the surfaces surrounding the opening are waxy. Hydrophobic forces between the watery secretion and the surface cause the secretion to form into a sphere. During secretion, these ovules have their micropyles facing horizontally or upward, i.e., not downward. The non-saccate pollen sinks through the drop, coming to rest inside the ovule (Tomlinson et al., 1997). By the time pollen reaches the nucellus, it is ready to germinate. The pollen tube grows and penetrates the nucellus. This mechanism occurs in *G. biloba* (Del Tredici, 2007), Cycadales (Schneider et al., 2002), Gnetales (Endress, 1996), and conifers, e.g., Cephalotaxaceae, Cupressaceae, Sciadopityaceae, Taxaceae, and the phyllocladoid species of Podocarpaceae (Tomlinson, 2012). An advantage of this PCM is that, depending on species, it serves as a key adaptation in both wind and animal pollination syndromes.

Understanding the constituents of this most prevalent PCM among extant gymnosperms is key to understanding the variety of roles that pollination fluids play in the reproductive biology of gymnosperms. We will look at water, sugars, amino acids, proteins, calcium and phosphates, as well as their role as nectar, and in pollen capture, delivery, germination, and in ovule defense.

##### Water

Water not only captures and hydrates pollen, but in many species, e.g., *Cupressus arizonica* (Chichiriccò et al., 2009), and *G. biloba* (Lu et al., 2016), water also causes pollen to shed its exine layer. This is an important event prior to germination. In cupressaceous conifers, exine shedding is also functionally significant. Removal of the hard-shelled exine layer, reveals the intine, which is a much more flexible layer. Additionally, exine-covered pollen is too wide to be able to enter via the ovule’s micropyle, but pollen with only intine present deforms easily, allowing it to squeeze through the narrow opening (Takaso and Owens, 2008).

Isolated pollen of PCM α species generally does not germinate in water alone. Pollen of *Taxus baccata* (Anhaeusser, 1953), *Ephedra gerardiana* (Mehra, 1938), and *E. aphylla* (Moussel, 1980) readily germinated in isolated pollination drops, but did not germinate in water controls. This rules out one of the first tempting ideas about pollination drops, namely that they replace simple rainwater. We can conclude that the first of the three functions of pollination drops–pollen capture–may be largely due to water, but the other functions, germination, pathogen defense, and nectar, depend on solutes.

##### Sugars

The most universal and abundant solute in these watery drops is carbohydrate. The three most important sugars are glucose, fructose and sucrose. These three make up over 95% of total sugar content (TSC). In a study of sugars in pollination drops, it was found that sugars other than glucose, fructose and sucrose make up less than 1% TSC. These include melezitose and xylose, as well as two sugar alcohols (Nepi et al., 2017).

Sugars in pollination drops are necessary for pollen germination and pollen tube nutrition (Nygaard, 1977), as well as for the nutrition of insect pollinators (Kato and Inoue, 1994). When TSC is analyzed, ambophilous species can be easily separated from species that are either solely wind-pollinated or insect-pollinated. Wind-pollinated species had a significantly lower TSC than ambophilous species. TSC ranged from 20 to 50 mg/mL in the pollination drops of wind-pollinated species, whereas TSC ranged from 110 to 900 mg/mL in those of ambophilous species (Nepi et al., 2017).

The universality of sugars in pollination drops implies that they were present among the ancestors of extant gymnosperms. Although analyses tend to report stable sugar compositions, in some species of *Gnetum*, sugar concentration can vary according to relative humidity. This is due to the high relative water content of the surrounding atmosphere, e.g., measurements of TSC of pollination drops of *G*. *gnemon* growing in a tropical rainforest ranged from 3 to 13% over the course of an evening (Kato et al., 1995).

##### Amino acids

All pollination drops have amino acids (Chesnoy, 1993). These include serine, glutamic acid, glycine, histidine, alanine and proline (Nepi et al., 2017). Just as sugar concentrations can be used to discriminate pollination drops of wind pollinated species from those of ambophilous species, the total amino acid content (TAC) of drops also proves to be a reliable predictor of pollination syndromes. Wind pollinated species have higher TAC values than ambophilous species such as *Gnetum gnemon*. From a nectar standpoint, it is not just a low total TAC that is important, but among the low concentration amino acids the relative concentrations of certain types of amino acids are significant also. One class of amino acids–non-protein amino acids–is characteristic of nectar. β-alanine, for example, may have desirable neurophysiological effects on insects that reinforce the role of nectar in attracting insects (Nepi et al., 2017). Concentrations of γ-aminobutyric acid, a suspected neurostimulant of insects, are very low if not zero in wind-pollinated gymnosperms, such as *Cephalotaxus* spp. (Chesnoy, 1993; Nepi et al., 2017).

##### Proteins

Proteins are found in all gymnosperm sexual fluids that have been analyzed to date. Because proteins are large complex molecules, by definition, they represent a sporophytic investment in the pollination drop that is substantial. This would be the case in what can be described as the “secretome,” i.e., proteins processed and secreted into the pollination drop by a tissue such as the nucellus. These proteins are thought to be active in the apoplast. However, some proteins are found in pollination drops as a consequence of cellular breakdown and are not normally found in the apoplast. This “degradome” is a consequence of nucellus cell death/breakdown to form a pollen chamber, for example in *Ephedra* spp. The degradome can be composed of over a dozen proteins (von Aderkas et al., 2015).

The most common proteins of the secretome include carbohydrate-modifying enzymes, such as glucanases, and defense proteins, such as anti-fungal enzymes, e.g., thaumatin-like proteins. These classes of proteins are nearly universal in pollination drops, which implies that they may have been there since the beginning of gymnosperm reproduction. As such, they represent a relatively well-preserved fraction of the functions of the pollination drop (Wagner et al., 2007).

Recently, proteomic analysis of pollination drops, coupled to a transcriptomic analysis of nucellus, was carried out on *Cephalotaxus koreana* and *C. sinensis* (Pirone-Davies et al., 2016). Pollination drops of these species have rich secretomes with nearly 30 proteins, many of which are involved in defense, carbohydrate-modification, or pollen growth. There are also a number of unique proteins that likely function in starch and callose degradation. This parallel gene expression study revealed a number of transcripts likely involved in pollination drop secretion, such as sugar transporters, β-glucosidases and P-loop-containing nucleoside triphosphate hydrolases.

In addition to such carbohydrate-modification and defense-related proteins just described, proteins have also been found that may play a role in regulating pollen growth and selection. Arabinogalactan proteins were found in pollination drops (O’Leary et al., 2004), which are involved in sporophytic selection of pollen tubes in angiosperms.

Protein composition of pollination drops of cycads, *Ginkgo* and many groups of conifers have yet to be studied. In addition, protein profiles, comparing male and female nectars found in strobili of the Gnetales need to be analyzed, as they may show differences as seen in angiosperms (Chatt et al., 2018).

##### Calcium and phosphate

Calcium is important for pollen germination (Brewbaker and Kwack, 1963). Recent studies have shown it to be present in *Ginkgo* pollen intine (Lu et al., 2016). Phosphate was identified long ago in pollination drops of *T. baccata* and *E. distachya (*Ziegler, 1959), but the form of phosphate was not established. Recently, we found evidence in a transcriptomic analysis of *Cephalotaxus* nucellus during pollination drop secretion of expression of a gene involved in eATP regulation – an apyrase (Pirone-Davies et al., 2016). Since phosphates, such as extracellular ATP (eATP), have immunogenic functions, including regulation of responses to fungal invasion in seed plants (Gust et al., 2017), pollination drops ought to be analyzed for their phosphate content.

##### Overall patterns in PCM α of nectariferous vs. non-nectariferous pollination drops

The strongest evidence that differentiates nectar from non-nectar pollination drops comes from the recent Principal Component Analysis (PCA) of carbohydrates and amino acids of ovular fluids (Nepi et al., 2017). PCA effectively separates out ambophilous from wind-pollinated species. The main factors in the clustering of the samples were; TSC (low in anemophilous species; high in ambophilous species), TAC (high in anemophilous species; low in ambophilous species), and non-protein amino acid percentage (low/absent in anemophilous species; high in ambophilous species). Absolute concentrations explained 70% of the variation. Ambophilous species overlap with flowering plant nectar (Nepi et al., 2017). In the PCA analysis, cycads, such as *Zamia furfuracea* that are beetle-pollinated (Norstog et al., 1986), clustered closer to the wind-pollinated conifer species, because of the low concentrations of sugar in their drops. However, a significant percentage the amino acids present was that of β-alanine, a rewarding compound for insects. Nepi et al. (2017) concluded that natural selection for strictly nutritional needs of these insects had had a lower impact on the chemistry of these cycad pollination drops. Chemical analysis also yielded a surprise: profiles of *G. biloba* pollination drops firmly placed this species among ambophilous species, namely those species for which nectar was a significant reward to insects. *G. biloba* is often referred to as wind-pollinated, e.g., Jin et al. (2012a), but as it is the last remaining species of what was once a species-rich clade, the PCA analysis would suggest that not just the surviving species of *Ginkgo*, with its high sugar concentration and non-protein amino acids, was once or still is, insect-pollinated, but that extinct ginkgophytes may have also been insect-pollinated.

What are the differences between a PCM α pollination drop and nectar? In our opinion, there are not many. In terms of evolution, the original pollination drop of the common ancestor of seed plants must have had at least the same four functions seen in extant species with PCM α: microgametophyte capture, delivery, germination, and ovule defense. Later, or possibly very early on, this drop acquired another function – insect reward. Such a pollination drop can be called either nectar or a pollination drop with a nectar function (Jörgensen and Rydin, 2015), but it is more expedient to focus on the ecological services, and call it nectar. The diversity of modern nectar types has resulted in nectar terminology being beset by historical circumstance (for discussion see Koptur, 1992). For example, angiosperm nectaries were the first to be divided into floral and extra-floral nectaries (EFNs), which has led to fern nectaries being referred to as EFNs, since they lack flowers. As Marazzi et al. (2013) point out in their survey of nectar-producing tissues, almost every above-ground part of flowering plants has been associated with nectar production. Nectar secretion processes are diverse enough to defy simple categorization based on anatomy. Nectar, it turns out, does not always flow from a nectary. Nectar is simply a sweet apoplastic fluid available on a plant surface where it can attract some animal or other that consumes it as a reward. Like many fern and angiosperm nectars, gymnosperm nectar does not, in the case of PCM α, originate from a nectary. Since it is of uniquely ovular origin, there is probably no modern angiosperm homolog. The nectar definition resides on the ecological service provided, that is, the mutualism of which it is a part. Nectar secreted by ovules of gymnosperms attracts many pollinators such as lizards (Celedón-Neghme et al., 2016), nocturnal moths (Kato and Inoue, 1994; Rydin and Bolinder, 2015), flies and wasps (Kato et al., 1995; Wetschnig and Depisch, 1999), even ants (Bolinder et al., 2016). It is the considered view of some nectar experts that pollination drops are functionally equivalent to angiosperm nectar (Bernardello, 2007).

Nectar production, when it is well-known, occurs in extant gymnosperms with PCM α, and thus far appears to be restricted to dioecious species. *Gnetum* spp., *E. foeminea*, and *Welwitschia mirabilis*, produce nectar from both male and female strobili (Nepi et al., 2017). In contrast, wind-pollinated species of *Ephedra* lack nectar production on their male strobili (Bolinder et al., 2016). In both the female and male strobili of *Gnetum* and *Welwitschia*, ovules produce drops that are sugar-rich and contain non-protein amino acids (Nepi et al., 2017). The largest difference between males and females is that the ovules in the male strobili are non-functional, sterile structures, the only function of which appears to be secretion (Haycraft and Carmichael, 2001). This is one of the unique aspects of nectar production among extant gymnosperms. It would be interesting to investigate gene regulation of ovule development to see whether ovules in male strobili are indeed different from those in female strobili. Because turning an ovule to another purpose is not common among plants, it would be of interest to know whether ovule development is redirected only for the purpose of providing nectar to attract insects. Nepi et al. (2017) found that male nectar had less volume, with lower TSC than female nectar. Compositional differences also exist. Fertile ovule secretions had greater fructose concentrations than those of male secretions. Higher concentrations of non-protein amino acids were found in fertile ovules than in male secretions. This is similar to results reported for male and female flowers of flowering plants. For example, in species of *Cucurbita*, male and female flowers of *Cucurbita maxima* ssp. *andreana* differ in their overall nectar production (Ashworth and Galetto, 2002), *C. pepo* male and female nectars differ in their sugar composition (Nepi et al., 2001), and *C. maxima* cv. Big Max male and female nectars differ in both metabolome and proteome (Chatt et al., 2018).

Nectar in male plants has two possible sources. The first source is pollination drops of the PCM α type, which produce a nectar in *G. parviflorum*, for example, which moths will search out with their probing proboscises (Kato et al., 1995). Moths will also search for any nectar that has seeped onto collars (Rydin et al., 2010). In some ways the situation is analogous to EFNs of plants such as *Acacia longifolia*, in which EFNs are in very close proximity to floral organs, which lack nectaries. Birds seeking nectar from EFNs unavoidably pollinate the flower (Thorp and Sugden, 1990). A second and more controversial nectar source in gymnosperms has been reported from male plants of *E. aphylla* (Bino et al., 1984). Here, nectar is non-ovulate in origin: it is produced from epidermal stomata of bracts of male cones. Although there are micrographs showing stomata and the sub-epidermal tissue of this nectary, the function of these nectaries has been called into question (Bolinder et al., 2016) and ought to be more closely investigated, as it is the only case of non-ovular nectar source known in any extant gymnosperm.

Once nectar is invoked, it raises several questions. There are a range of insect behaviors that must be considered. Is a pollination drop still nectar if it only occasionally feeds opportunistic insects, only minimally contributing to reproductive success? Opportunistic nectar feeding by a broad range of insects, including those that are not considered pollinators (i.e., ants), has been described by various authors (Little et al., 2014; Bolinder et al., 2016). Another question concerns whether a pollination drop is still nectar if it attracts parasitic insects that do not contribute at all to the reproductive success of the plant. Chalcid wasps that parasitize ovules are attracted to pollination drops of *Ephedra* (Moussel, 1980; Bolinder et al., 2016). In addition to parasitizing the ovules, these wasps feed on pollination drops also. Furthermore, the wasps can be present in sufficient numbers that they consume the majority of drops produced by ovules in the local plant populations. After sucking up the pollination drops, the insects oviposit their eggs into the ovule (Moussel, 1980). At first glance, one would expect that a seed parasite such as a chalcid wasp would be ruining its own opportunities by depressing the plant’s ability to set seed, but these parasites are able to alter megagametophyte metabolism in such a way that the ovule – in spite of its reproductive failure – fills with the very reserves its embryo would require, only now they are solely available to the parasite (Favre-Duchartre, 1960). In this case, the nectar is only the first in a series of high energy substances that the parasite uses for its own offspring. In this, nectar-producing gymnosperms are victims just as much as non-nectar producing gymnosperms. For example, the chalcid wasp *Megastigmus spermotrophus*, a seed predator, parasitizes megagametophytes of *Pseudotsuga menziesii*. By injecting venoms, the chalcid may be redirecting the megagametophyte’s metabolism (Paulson et al., 2016).

There are other aspects of nectar that await study in gymnosperms. For example, if we look to angiosperm nectar, a diversity of secondary metabolites has been found that affect the interactions between plants and their pollinators (Roy et al., 2017). In gymnosperms, analyses are lacking for a number of classes of metabolites, including lipids, phenolics and terpenoids that might be present in gymnosperm nectar. Another aspect of gymnosperm nectar that warrants at least preliminary study is a possible nectar microbiome. For example, a number of angiosperm species have been discovered harboring yeasts in their nectar (Nepi, 2017). It is not unreasonable to expect a microbiome in these nectars that are exposed to the environment and have complex plant-animal interactions.

#### Pollen Capture Mechanisms β and γ

The remaining PCMs differentiate themselves from PCM α in morphology, behavior and chemistry. In our simplified classification of PCMs, we present two other basic types of PCMs, β and γ that relate to both primary pollen capture surface and clade. PCM β represents a diverse set of pollination mechanisms found in Pinaceae in which the primary pollen capture surface is the integument. Many of these use lipid-based microdrops as part of this primary capture (Figures 4C–I). The clade comprised of Podocarpaceae and Araucariaceae (Figure 1) possesses PCM γ (Figures 4J,K). Generally, in these, extra-ovular surfaces capture pollen. Some phyllocladoid podocarps use drops for pollen capture similar to PCM α, but in these cases the pollen either lack sacci or have vestigial/non-functional sacci (Tomlinson, 2012). This is an interesting parallel with one pinaceous species, *Picea orientalis*, in which sacci have become non-hydrodynamic and ovules remain upright at time of pollen receptivity (Runions et al., 1999). Currently, it is thought that all species with PCM β and PCM γ are anemophilous. However, there is compelling recent evidence that ancestors or extinct sister-groups of these clades may have been ambophilous in the Mesozoic (Labandeira et al., 2007; Ren et al., 2009; Labandeira, 2010). Consequently, the biochemical profiles of these PCMs are of immediate importance in any discussion of ancient nectar production in a clade that has seemingly lost that capacity.

There are a number of ways in which PCMs β and γ differ from PCM α. In PCM β, found among Pinaceae (e.g., *Picea, Pinus*), lipid microdrops are secreted from two integument extensions, or flaps, at the tip of the ovule. Pollen adheres to these microdrops. An ovular pollination drop is then produced, which removes the pollen from the integuments. Since the pollen of these species is saccate, a morphological feature that confers buoyancy, the pollen floats upward into the ovules (Owens et al., 1987). There is a selective element to this, as saccate pollen is preferentially taken up compared to non-saccate pollen (Leslie, 2010). The ovules of species with saccate pollen are characteristically inverted at the time of pollination. This is in contrast to PCM α where non-saccate pollen sinks into the drops of more or less upright ovules. A variant of the pinaceous PCM β seen in *Cedrus* has pollen captured by microdrops on an irregular funnel shaped integumentary margin, with a drop arriving later to deliver pollen into the ovule (Saxton, 1930; Takaso and Owens, 1995a,b). *Pseudotsuga* and *Larix*, have non-saccate pollen that is trapped by sticky, terminal integumentary hairs. These hairs collapse inward, which physically delivers the pollen into the ovule interior. Many weeks or a few months later a drop is secreted (Takaso et al., 1995) that brings the pollen to the nucellus surface (Takaso and Owens, 1997), and germination is triggered (Villar et al., 1984; Said et al., 1991). This drop has been called a post-pollination prefertilization drop. It is smaller than a PCM α pollination drop and fills the volume of the micropyle only (Figures 4G,H). *Abies* appears morphologically similar to *Cedrus*, but is thought to lack a pollination drop (Owens and Molder, 1977; Chandler and Owens, 2004).

In the case of PCM γ, which is restricted to Podocarpaceae (Tomlinson, 1991, 1994), neither the tips of the micropyle, nor the surrounding surfaces of the bract are coated with wax. Subsequently, the pollination drop is not hemispheric, but assumes a spreading amorphous form that scavenges pollen from a larger area than is possible with PCM α (drop capture) or PCM β (integumentary capture). In some species, an ovule may repeatedly secrete and withdraw its pollination drops. Similar to the pinaceous PCM β, pollen are saccate, and ovules inverted. Again, pollen entry into the ovule is due to flotation. However, there are no known surfaces with lipid microdrops (i.e., PCM β) as part of primary pollen capture in PCM γ.

##### Sugars

Analysis of pollination drops from species with PCMs β and γ has not been done in any broadly sampled, systematic way. However, carbohydrate concentrations have been reported from pollination drops of several taxa of the pinaceous PCM β, including *P. engelmannii* (Owens et al., 1987), *Pinus nigra*, and *P. resinosa* (McWilliam, 1959), which all have low TSC, i.e., concentrations are generally less than 5%. Among sugars, fructose dominates: there is little glucose and generally no sucrose. Polysaccharides such as galactose, arabinose, rhamnose and mannose are often detected, but at low concentrations (Chesnoy, 1993). Drops of *Pseudotsuga menziesii* also have similarly low concentrations of these carbohydrates, whereas *Larix* x *marschlinsii*, (in the genus sister to *Pseudotsuga*), has a relatively high concentration of sucrose, e.g., 53 mg/mL (Nepi et al., 2017). Differences in the TSC between these species is thought to be responsible for the differential responses of pollen that were observed after application of cross-generic pollen (von Aderkas et al., 2012). No sugar concentrations from species with PCM γ are as yet known.

##### Amino acids

The profiles of amino acids in PCMs β and γ, where known, are typical of wind-pollinated species. Amino acids include serine, aspartic acid, glutamate, proline, glycine, α-alanine, and traces of others, such as leucine, isoleucine, threonine, glutamine, aspartate. Non-protein amino acids, such as β-alanine that are present in nectar of all ambophilous species with PCM α, are almost completely lacking in taxa that have PCMs β and γ (McWilliam, 1959; Nepi et al., 2017).

##### Lipids

Lipids also appear commonly as microdrops on integumentary extensions of *Picea* (Owens et al., 1987), *Pinus* (Owens et al., 2001), and *Cedrus* (Takaso and Owens, 1995a). Unfortunately, no chemical analyses of these integumentary lipid secretions have been made to date.

##### Proteins

The only species that have been studied outside of PCM α are *L.* x *marschlinsii* and *P. menziesii* (PCM β). Just as in PCM α, defense proteins such as chitinases (Coulter et al., 2012) and thaumatin-like proteins (O’Leary et al., 2007) were identified. In addition, the *in situ* activity of these enzymes has been confirmed. Carbohydrate-modifying enzymes have also been found, including xylosidases, galactosidases (Poulis et al., 2005), and invertases (von Aderkas et al., 2012). Serine carboxypeptidase, peroxidase, and aspartyl protease were detected (Poulis et al., 2005). In summary, many of the same enzymes involved in ovule defense and carbohydrate-modification are found across all gymnosperms, implying a conserved ancestral function. However, this conclusion is based on two species and begs further investigation of PCM β. Species of PCM γ remain unsampled for proteins.

##### Calcium and phosphates

Calcium is abundant in post-pollination prefertilization drops of *Larix* and *Pseudotsuga* (von Aderkas et al., 2012). Phosphate compounds await investigation. Again, this is likely to be conserved among gymnosperms, but further study is needed for confirmation. Given that both of these compounds are well-known and important in angiosperm pollination biology, investigation of these compounds represents a key gap in our knowledge.

##### Overall patterns in PCM β and γ of nectariferous v non-nectariferous drops

Species with PCMs β and γ are not involved in nectar production today, but according to Ren et al. (2009) ancient members of these might have had insect pollinators. In particular, PCM β has been discussed in relation to insect pollination in the Mesozoic (Ren et al., 2009). Given that today there are no such insect-pollination drop relations among extant species with PCM β, how is this possible? An interesting possibility is already available in the case of species such as *L.* x *marschlinsii*, which has higher sugar concentrations (∼100 mg/mL) compared with other conifers. The recent analyses of Nepi et al. (2017) adds support for the idea that in the Mid-Mesozoic there may have been conifers that produced a passable nectar and could have been insect-pollinated, specifically by long-proboscid scorpion flies (Peris et al., 2017). This implies that a trait such as total sugar concentration (TSC) in pollination drops may be under natural selection, and as a result, insect pollination mutualisms are more likely than previously thought. It is certainly within the realm of possibilities, because recent phylogenetic analysis of *Ephedra* provides evidence that in at least one gymnosperm clade pollination syndromes evolved from the plesiomorphic state of insect-pollination to wind pollination (Bolinder et al., 2016). If it could have happened in the gnetalean *Ephedra*, could it also have occurred in ancient Pinaceae? For example, similar reversion from insect to wind pollination is common in angiosperm species, where it has occurred as many as 60 times (Koptur, 1992). More *direct* observation of insect pollinators is required. Because insect pollinator communities thrive in ecosystems that provide resource diversity, as pointed out in Saunders’ (2018) meta-analysis of insect pollinators collecting pollen from wind-pollinated plants (including Pinaceae), it is not surprising that even a little bit of carbohydrate-supplemented fluid probably goes a longer way in attracting insects than previously thought. Given that modern insects visit anemophilous species for pollen nutrition (Saunders, 2018), and that simple changes in regulation of invertase gene expression genes, as is also known to occur in flowering plants, results in changes in sugar concentration (Heil, 2015), we suppose that shifts from insect to wind pollination in gymnosperms may be more likely than previously thought.

## Fossil Gymnosperms

Integrating information from modern gymnosperm ovular fluids with the fossil record presents a challenge. To further our understanding of the origins of sexual fluids in seed-plants we must rely on a synthesis of data from modern plants with inferences based on morphological and anatomical fingerprints of biological function in the context of current phylogenetic hypotheses. By way of example, the earliest cycads, the crown group of which dates back to the mid-Permian (265 Mya) (Condamine et al., 2015), likely reproduced in a manner identical to how they reproduce today. Their conserved ovular features imply as much, even though direct fossil evidence of sexual fluids may be lacking. Direct observation of sexual fluids is expected to be rare precisely because of their ephemeral nature, but not impossible. Certain fossil localities with exceptional preservation (Lagerstätten) have shown rare cases of preserved plant exudates, e.g., mucilaginous plugs in the aroid seed, *Keratosperma*, from the Eocene Princeton Chert locality (Smith and Stockey, 2003). A permineralized pollination drop that contains prepollen is known from a callistophytalean from the Carboniferous (Rothwell, 1977). Much of the fossil evidence supporting a long history of sexual fluids is not based on direct discovery of preserved pollination drops, but on sound inferences made from anatomical fingerprints related to gymnosperm reproduction (Stewart and Rothwell, 1993).

### Timeframe

**FIGURE 5:**
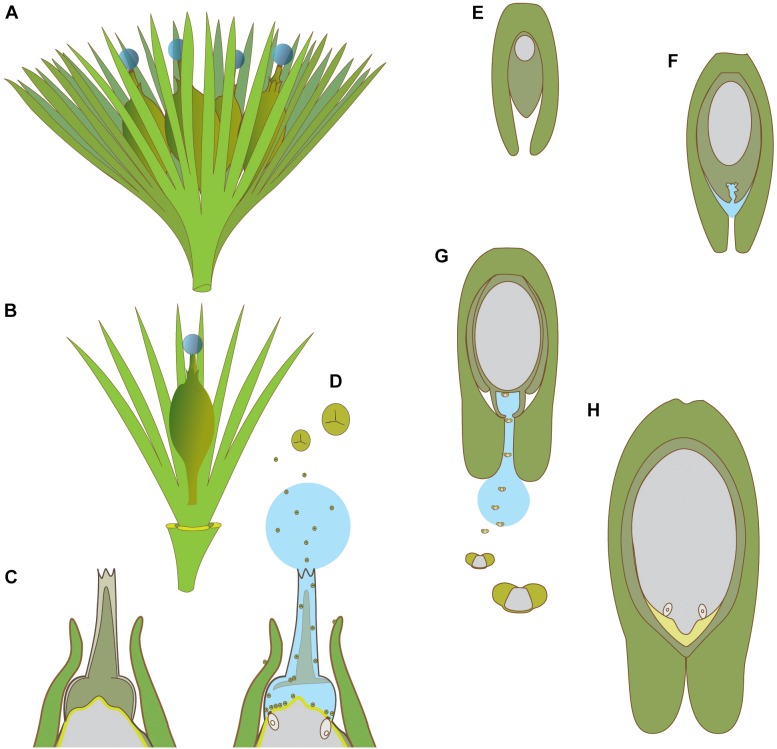
Schematic diagrams of pollination in hydrasperman ovules that appeared in the Devonian **(A–D)** and callistophytalean ovules that appeared in the Pennsylvanian **(E–H)**. **(A)** Cupule bearing four ovules with pollination drops. **(B)** Cupule quarter showing one ovule; three other cupule segments removed. **(C)** Longitudinal section through apex of ovule prior to pollination; megagametophyte (center, light gray) is developed, apical region of nucellus (dark gray) with tissue filling the salpinx (central column). **(D)** Longitudinal section through apex of ovule at time of pollination; central column tissue degrading (inferred PCD, see Figures 4A,C,D for examples in extant species), pollination drop present with trilete prepollen falling through the pollination drop; megagametophyte and archegonia exposed to prepollen. **(E)** *Callospermarion* (Callistophytales) ovule showing early stage with developing megagametophyte (center, gray) within the nucellus (dark gray). **(F)** Later stage with nucellus beginning to break down (inferred PCD), and pollination drop is forming. **(G)** Pollination stage with well-developed pollen chamber; pollination drop collecting saccate *Vessicaspora*-type saccate pollen grains that float up the drop to the nucellus surface where they germinate to produce haustorial pollen tubes, similar to those seen in extant cycads. **(H)** Fertilization stage with archegonia formed; apical chamber filled with fertilization fluid for swimming sperm (microgametophytes not shown). **(A–D)** based on Matten et al. (1980, 1984), Rothwell and Wight (1989), Serbet and Rothwell (1992), Erwin et al. (1994), Hilton and Bateman (2006), Galtier et al. (2007), Prestianni and Gerrienne (2015). **(E–H)** based on Rothwell (1971), Rothwell (1977).

The earliest fossil records for gymnosperm reproduction date from the Devonian. A megasporangium/nucellus (Figure 5) is surrounded by axes, or laminar organs, borne on structures called cupules (Stewart and Rothwell, 1993). Homologies drawn between modern ovules and these preovules have been the source of much discussion in paleobotanical studies (Taylor and Millay, 1979; Leisman and Roth, 1984; Meyen, 1984; Hilton and Bateman, 2006). Generally, the structures surrounding the megasporangium have been called integumentary lobes, because they are considered to be homologous to the single gymnosperm integument (Taylor et al., 2009). The retention of a megasporangium on the sporophyte is called the seed-habit, which is defined, at least in part, by whether embryos mature on the sporophyte, and by where integuments form the micropyle (Herr, 1995).

Supporting arguments for ancient origins of sexual fluids come from studies of microgametophytes (prepollen and pollen), and both preovules and ovules. The presence of liquids is often implied if a structure described from fossils is similar to a fluid secreting/vectoring structure known to function during reproduction. The examples provided below include prepollen, sperm, the functional requirements of saccate pollen, and the adaptations for pollen and/or sperm delivery such as channels for fluid-based pollen delivery and signatures of fluid production, such as cellular break-down in pollen chambers. At the end of this section we will also touch on the kind of fossil evidence for nectar.

#### Prepollen

Prepollen characterizes virtually all Paleozoic gymnosperms (Poort et al., 1996). Having a proximal aperture similar to that found in modern free-sporing heterosporous plants is an indicator of endosporic microgametophyte development and release of swimming sperm from the aperture at maturity (Chaloner, 1970; Rénault, 1887). Prepollen is thought to have germinated proximally, via the monolete or trilete meiotic groove as in free-sporing plants. Observation of ephemeral free swimming sperm in fossils is understandably rare (but see Benson, 1908; Stewart, 1951; Nishida et al., 2003). The transition from prepollen to modern pollen has been studied, although it is not clear for all groups, for example, Cordaitales and voltzialean conifers have either prepollen, or modern-looking pollen with a distal aperture, and in some instances both (Gomankov, 2009). It is possible that many extinct taxa had a transitional type of microgametophyte development between prepollen and modern siphonogamous pollen, similar to that seen in cycads and *Ginkgo* in which the pollen tube germinates distally to produce haustorial tubes, penetrates the nucellus, and develops later to release swimming sperm proximally. Haustorial pollen tubes have been observed in Callistophytales (Rothwell, 1977; Figures 5E–H). Preserved spermatozooids within microgametophytes inside the apices of ovules have been documented for a glossopterid (Nishida et al., 2003, 2004, 2007). Taken together, the presence of prepollen allows us to infer the presence of archegonial fertilization fluids. This is further supported by the preserved archegonial chambers – the site of sperm delivery – in the ovules of seed ferns such as *Lagenostoma* (Taylor and Millay, 1979).

#### Hydrasperman Anatomy

Modern gymnosperms and early seed plants have similar-looking pollen delivery channels. In modern gymnosperms the micropyle is formed by the integument; this tube is the pathway for sexual fluids for the direct or secondary capture of pollen, which later germinates to produce pollen tubes that penetrate the nucellus (see section above on extant PCMs α, β, and γ). In the earliest seed plants, there is a micropyle analog, formed from the apex of the megasporangium, or nucellus, called a salpinx (Figures 5C,D; Matten et al., 1980, 1984; Rothwell and Wight, 1989). Hydrasperman prepollen-receiving anatomical structures have been interpreted and labeled in different ways (see discussion by Hilton and Bateman, 2006). Prepollen is found within the salpinx in anatomically preserved fossils (Matten et al., 1980). Seeds appearing later in the fossil record maintained a modified version of this hydrasperman apical modification, including the members of the Lyginopteridales and the Medullosales (Meyen, 1984; Doyle, 2008). Thus, one of the interpretations is that similar shapes used by modern gymnosperms for pollen capture by a sexual fluid, i.e., a PCM α-type drop, were probably present in these extinct plants.

#### Saccate Pollen Grains

In modern gymnosperms, saccate pollen are a hydrodynamic adaptation in which the hydrophobic nature of the pollen wall allows the pollen grain sacci to inflate upon contact with the pollination drop (Salter et al., 2002). Sacci and inverted ovules are another anatomical fingerprint for drop delivery at the time of pollen receptivity. Sacci provide buoyancy for the grain, which is then able to float upward in the drop through the micropyle to the nucellus, where the pollen germinates (Doyle, 2008; Leslie, 2008, 2010). A notable example from the fossil record is in the saccate glossopterids (Nishida et al., 2003, 2004, 2007). Their cycad-like microgametophytes, which have been found preserved in the apex of fossil seeds, have mature sperm cells just prior to release. Among conifers, the developmental link between saccate pollen and pollination drops is of considerable importance in the evolution of conifer pollination mechanisms (Leslie et al., 2015). It is interesting to note that saccate pollen is prevalent among many extinct gymnosperms lineages, including Peltasperms, Corystosperms, Callistophytales, Cordaites and Voltizales *sensu lato* (Doyle, 2010; Bomfleur et al., 2013).

#### Nucellar Degradation, Pollen Chambers and Micropyles

In many gymnosperms, pollination drop secretion coincides with breakdown of apical nucellar tissue (Singh, 1978), presumably by programmed cell death (PCD). In cycads, *Ginkgo*, Gnetales, and some Pinaceae, cells degrade to form a chamber (Figures 4A,C,D). Protein profiles of these drops show the expected signature of a degradome that is predicted for a PCD-derived exudate (von Aderkas et al., 2015). Virtually all Paleozoic fossil ovules, e.g., hydraspermans, Lyginopteridales, Medullosales, that are anatomically preserved show some degree of apical nucellar cellular breakdown to form (pre)pollen chambers. The earliest seeds with anatomical preservation show signs of PCD during pollination (Figures 5C,D,F; Rothwell, 1971; Matten et al., 1980). Signs of PCD in fossil nucellar apices provides another anatomical fingerprint for the presence of pollination drops.

#### Presence of Prepollen and Pollen in Pollen Chambers

It is unlikely that significant numbers of prepollen or pollen could accumulate by chance and gravity alone into the pollen chambers of ovules. As in modern gymnosperms, some mechanism must have existed to increase efficiency. In modern gymnosperms, the drop captures directly (PCM α) or scavenges secondarily from integumentary (PCM β) or extra-ovular surfaces (PCM γ) to bring pollen into the interior of the ovule (Tomlinson et al., 1997). In hydraspermans, prepollen grains are often found in anatomically preserved ovules (Taylor et al., 2009). Where integumentary lobes are short, i.e., around the ovule, salpinxes are reduced. This suggests the extension of the salpinx, a structure for capturing pollen, is to optimize exposure of the drop to the environment for prepollen capture. Niklas (1983, 1985) shows that preovules with integumentary lobes close to the salpinx had greater numbers of simulated prepollen capture events, although this includes several other factors, such as orientation of the preovules. The long micropyles with pollination drops of modern-day Gnetales function similarly to capture pollen (El-Ghazaly et al., 1998). Fluted, tubular, apical micropylar structures bearing pollen grains in their base are common in anatomically preserved fossil ovules. It has been argued that increasing the distance that microgametophytes and their gametes have to travel to achieve fertilization represents a trend of increasing sporophytic control of microgametophyte development (Lora et al., 2016).

#### Nectar

Whether pollination drops in fossil gymnosperms functioned as nectar is not clear, although the Lyginopteridales show some early evidence for insect interactions based on the presence of glands on both vegetative and reproductive structures [Oliver, 1909; reviewed by Labandeira et al. (2007)], which today often function in plant-animal interactions. There is better support for this later in the fossil record, e.g., Medullosalean prepollen grains were too large for wind pollination (Schwendemann et al., 2007). A consensus for Mesozoic insect pollination has been growing with mounting evidence based on new insect and plant fossils (Ren et al., 2009; Labandeira, 2010). According to Labandeira et al. (2007), early pinopsids such as Cheirolepidiaceae have structural modifications that are suggestive of insect pollination, implying that insects were attracted by pollination drops. Since nectar formation in modern gymnosperms is not associated with obvious nectaries, but is a nucellar product, the anatomical fingerprint is the nucellus. The basis for believing that nectar was possible in the past is based on the range of sugar concentrations that can be produced by the modern gymnosperm nucellus. Sugar concentrations in insect-pollinated modern gymnosperms are similar to those of insect-pollinated angiosperms; even wind-pollinated conifers produce, depending on species, a broad range of carbohydrate concentrations (Nepi et al., 2017). There is no reason to assume that such flexibility in carbohydrate production by ovule nucellus could not have existed in the past. Additional support for the presence of a PCM α style drop comes from wind pollination experiments performed on models of several early seed plants (Niklas, 1983). Several species were shown to have relatively inefficient wind-based capture based on their morphology. Given caveats, it must be plausible to consider that some of these earliest seed-plants could have had animal-assisted pollination.

## Drop Dynamics

A number of physiological characteristics of pollination drop behavior contribute to wind and insect pollination syndromes in modern gymnosperms. Some of these elements are of importance in imagining how the early gymnosperms described in the previous section reproduced. Understanding drop dynamics is also important if we are eventually to understand nectar dynamics.

**FIGURE 6:**
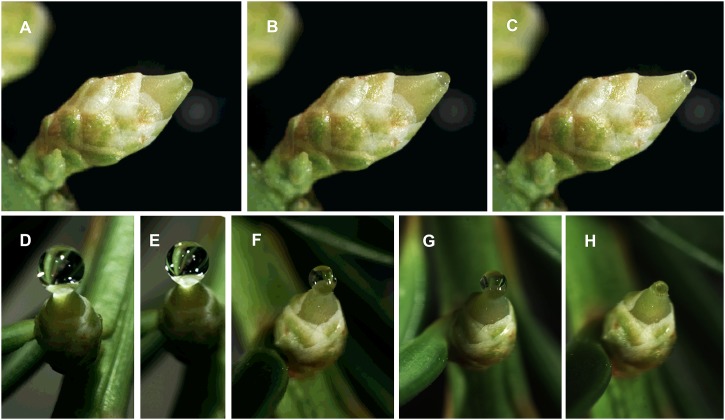
Photographs from time-lapse study of *Taxus* x *media* pollination drop activity; photos by S. Gagnon. The series show pollination drop reformation after removal of initial drop: **A** time 0; **B** 50 min; **C** 100 min. The series show pollination drop retraction: **D** time 0; **E** 1 h, when pollen was dusted onto the drop and ovule using a syringe; **F** – 5 h; **G** – 20 h; **H** – 23 h.

On the surface of it, drop behavior appears to be simple: a pollination drop is secreted, and sooner or later, it retracts (Figure 6). Drops form prior to pollination and retract when they are pollinated by wind or insect, with the exception of some Pinaceae (Owens et al., 1998). Most of the information that we have on behavior is based on pollination drops that are readily accessible and easily viewed, such as those of PCM α species. Species in which ovules are deep within strobili and hidden from view are more difficult to study, e.g., Pinaceae, Taxodiaceae and Sciadopityaceae (Tomlinson, 2012). In this section, we will consider plant behavior in terms of the pollination drop functions of pollen capture and germination. Some examples of species for which we have nectar-specific information with respect to capture and germination will be discussed also.

### Pollination Drop Secretion and Retraction

Pollination drop formation in various species either occurs prior to pollination (PCM α, non-phyllocladoid γ) or after (PCM β). Pollination drops originate in the nucellus, as shown by immunolocalization studies of pollination drop proteins (Poulis et al., 2005).

#### Regulation of Secretion

The secretion period may vary according to pattern and length. Secretion is often considered to be diurnal in nature (McWilliam, 1959; Strasburger, 1871), but as more phenological studies are carried out, an appreciation of the complexity of secretion has developed. Some species secrete their drops only during the day, e.g., *Cephalotaxus* spp., podocarpaceous conifers (Tomlinson et al., 1991), *Z. furfuracea* (Tang, 1987). Other species produce drops at night, such as those of nocturnally pollinated species of *Ephedra* (Rydin and Bolinder, 2015), and *Gnetum* (Kato et al., 1995). Unpollinated drops may last many days before retracting, e.g., 5 days in the case of *Taxus chinensis* (Xing et al., 2000) and up to 12 days in *Juniperus* (Mugnaini et al., 2007b). According to Dörken and Jagel (2014), pollination drops of cupressaceous conifers are present both day and night. There is no evidence of diurnal rhythms in secretion and retraction for the over twenty species that they investigated. In contrast to this apparent absence of a diurnal pattern is the example of a cupressaceous conifer with a far more complex pattern in which diurnal secretion is only one part of a longer pattern that spans days. In *Chamaecyparis nootkatensis*, drops are secreted during the night and then retracted the next day. This pattern is repeated for the first few days of the pollination period, but then a drop is secreted that lasts for many days and nights without retracting, before its final retraction ends the pollination period (Owens et al., 1980). Cones of *L.* x *marschlinsii* produced post-pollination prefertilization drops in rhythms that were independent of the diurnal water potential patterns of the trees to which they were attached, which led to the conclusion that at least in some species the regulation of secretion is controlled at the cone and even the ovule level (O’Leary and von Aderkas, 2006).

These basic secretion patterns also occur together with other aspects of pollination syndromes. Insects are attracted by rewards and by smell. In *G. gnemon*, which has an ambophilous pollination syndrome, nocturnal moths are attracted by putrid volatiles released from strobili. *G. cuspidatum* attracts nocturnal flies with smells that recall rotten wood and fungi (Kato et al., 1995). *G. gnemon* secretes its pollination drops in the early evening in concert with the release of volatiles. The drops are retracted in the early morning. This pattern repeats itself. In insect-pollinated *Macrozamia* species, female strobili release volatiles at specific times of day that are coordinated with thermogenesis as part of the complex ‘push-pull thermogenesis’ system that controls pollination (Terry et al., 2007).

#### Regulation of Retraction

Retraction of pollination drops is governed by internal ovule physiology, external factors such as atmospheric evaporative demand and presence of pollen. Ziegler (1959), who studied *Ephedra* and *Taxus* concluded that retraction was strictly regulated by evaporation. In contrast, Tomlinson (2012) noted that the interaction between pollen and pollination drop was a more hydrodynamic process, triggering other processes that influenced water availability. Pollen enters the ovule, the micropyle of which rapidly seals shut, preventing further contamination by foreign pollen or microorganisms. Both pollen capture and ovule defense operate in concert with one another (Tomlinson, 2012). The entire ovule appears to be involved with retraction of pollination drops, as absorption experiments have shown using either Acid Fuchsin (Tison, 1911) or colchicine (Favre-Duchartre, 1958).

A study involving *Juniperus oxycedrus* (Mugnaini et al., 2007a) provides different evidence for retraction as a two-step process. As in the previous examples of cupressaceous given above, pollen of *J. oxycedrus* hydrates, loses its exine, triggering drop retraction. However, Mugnaini et al. (2007a) also found that foreign particles (beads, dust, foreign pollen [i.e., non-cupressaceous pollen]), as well as non-viable homospecific pollen caused an initial small diminution of the pollination drop, which was only followed by complete retraction if the pollen was of a cupressaceous species. This is an interesting result, as it should be recalled that unpollinated *Juniperus* pollination drops remain unretracted for up to 12 days, but once pollinated, retract in just minutes. In essence, this prevents entry of foreign objects into the ovule, which again points to pollination drops playing a role in ovule defense.

Pollen may also affect retraction in other ways. Xing et al. (1999) removed pollination drops from one cupressaceous species only to replace them with pollination drops from another cupressaceous species. The “replacement drops” receded when pollinated, but took much longer for complete withdrawal. However, the rate of retraction could be increased in proportion to the number of pollen grains added. The authors stated that pollination drop withdrawal is due to pollen regulation of the secretion process. This points to an effective recognition system for pollen by the ovule, possibly mediated via the nucellus. Further support for a recognition system comes from a comparison of retraction rates of pollination drops dusted with pollen sourced from evolutionarily close species to retraction rates of pollination drops dusted with pollen from distant species (Dörken and Jagel, 2014). The closer the evolutionary distance of the pollen, the faster the retraction response of cupressaceous pollination drops. However, it is not clear what the advantages of speed are. These sporophyte-gametophyte interactions, i.e., between nucellus and pollen, appear to carry a cost. The advantage of such a rapid retraction belies the lack of discrimination. Once pollinated, cupressaceous conifers do not initiate a new secretion, which means that capturing closely related but “wrong” pollen results in inevitable reproductive failure.

#### Pollination Drop Replacement

An important question in pollination drop physiology is drop replacement. Rain, sudden movement, and high evaporative demand can cause drops to disappear or be removed. In the case of nectar, non-pollinating insects can remove drops. In all of these cases, gymnosperm reproduction would be brought to a standstill if drops could not be replaced. If the one-and-only drop fails to collect pollen, then no other drop is produced and reproduction would fail. However, many species have drop replacement. *Thujopsis dolobrata* pollination drops can be replaced a maximum of eight times in succession (Dörken and Jagel, 2014). In short, the loss of a given drop does not lead to loss of function of the ovule, as it is able to replace the drop. In insect-pollinated species, replacement of drops is an important consideration, as the secretion that follows removal by an insect must play a role in scavenging pollen left at the rim of the micropyle by the pollinator. *E. aphylla* continues to produce pollination drops after pollen has already been captured from insects by an earlier drop (Moussel, 1980).

#### Pollination Drop Volume

Another aspect of pollination drops that has a bearing on pollination syndromes is drop volumes. Micropyle volume varies in species that have been measured, e.g., *P. menziesii* (Takaso and Owens, 1996b; von Aderkas and Leary, 1999a) and *L. x marschlinsii* (von Aderkas and Leary, 1999b). Of greater biological importance is the fact that pollination drop volumes vary between species. Insect-pollinated species in which pollination drops are functioning as nectar have much larger drops than insect-pollinated species in which only pollen is the reward. For example, pollination drops of *Gnetum*, a group that uses nectar as its primary reward, are in the 150–200 nL range (Kato et al., 1995), whereas pollination drops of cycads, a group that uses pollen as its primary reward, have volumes an order of magnitude less (Prior, 2014). Pollination drops of wind-pollinated species have small volumes (20–100 nL). There are some exceptions, such as *Taxus* spp., which have drops around 250 nL in volume (Nepi et al., 2009). There is also another type of exception, one that is particular to cupressaceous conifers. *Fitroya cupressoides, Cupressus sempervirens* (Dörken and Jagel, 2014), and *Chamaecyparis nootkatensis* (Owens et al., 1980) have cones in which the ovules are arranged so close to one another that synchronously secreted pollination drops fuse to form large amorphous drops. It has not been tested whether these ‘super-drops’ provide any advantages in pollen delivery efficiency or reproductive success.

Nectar viscosity may have an additional influence on insects. In *Gnetum*, nectar produced by sterile ovules on male strobili has a relatively low viscosity (Nepi et al., 2017) and tends to run and seep onto other structures, such as collars (Rydin et al., 2010). Insects are attracted to both the pollination drops and the run-off of these drops (Kato et al., 1995; Rydin et al., 2010). It would be worth testing whether the additional location of the nectar attracts nectar-seeking pollinators for a longer period, thereby contributing to greater reproductive success. Modeling micropyle and pollen chamber volumes may also be important for inferences of fossil plant biology.

#### Speed of Retraction

The speed of retraction varies. In *Taxus*, retraction following pollination takes 24 h (Figures 6D–H). Such a slow drop retraction may be entirely caused by evaporation (Xing et al., 2000). *Ginkgo biloba* is faster, taking only 4 h (Jin et al., 2012b). This slightly speedier process in *Ginkgo* is not solely caused by evaporation, but may also involve some undisclosed active process (Jin et al., 2012b). Active processes are thought to occur in two steps, the first of which involves pollen hydration and loss of its exine. The next step–active retraction–occurs as the pollen sinks into the drop (Lu et al., 2016). One family of conifers–Cupressaceae– is noteworthy in the rapidity with which pollination drop retraction takes place following pollination. Previous researchers had noted that species such as *Cephalotaxus drupacea* (Chesnoy, 1993), *Platycladus orientalis, Thuja occidentalis* (Xing et al., 1999), and *Chamaecyparis nootkatensis* (Owens et al., 1980) took less than 20 min. A broad survey in which pollination drop retraction times were measured in a few dozen cupressaceous species in response to pollen of another cupressaceous species, *Thujopsis dolobrata*, showed that retraction occurred, on average, in less than 10 min (Dörken and Jagel, 2014).

#### Nectar Retraction

In some species, pollination drops that act as nectar retract in response to pollination. *G. biloba* (PCM α), a putatively insect-pollinated species (Nepi et al., 2017), retracts its drop with a definite finality following pollen capture (Xing et al., 2000), but, as mentioned previously, other species with PCM α, such as *E. aphylla*, are able to produce pollination drops repeatedly following successful pollination, as well as after removal of the drops by insects (Moussel, 1980).

### Pollen Germination

Pollination drops induce germination of pollen *in situ*, e.g., *Ephedra* (Moussel, 1980), *Pinus* (McWilliam, 1959) and *in vitro*, e.g., *Ephedra* (Mehra, 1938; Moussel, 1980) and *Taxus baccata* (Anhaeusser, 1953). Pollination drops deliver pollen to the nucellus, where it germinates, e.g., *L. decidua* (Villar et al., 1984), *Cephalotaxus drupacea* (Chesnoy, 1993). The nucellus is a complex tissue from a secretion standpoint. In a developmental study of the nucellus of *C. drupacea* over the course of pollination drop secretion, it was noted that at the beginning glyco-proteins and polysaccharide substances were released from the nucellar apex, and that at the end proteins and lipids were secreted (Seridi-Benkaddour and Chesnoy, 1991). The early substances, those secreted during pollination, influence pollen development. In studies of intergeneric crosses, pollination drops induce germination of homospecific pollen, whereas heterospecific pollen germination is less successful, as was shown in a study of intergeneric crosses of *Larix* and *Pseudotsuga* (von Aderkas et al., 2012). That is not always the case, as pollen of any given *Pinus* spp. will readily germinate in the ovule of any other species: selection becomes obvious only as tubes begin to grow inside the nucellus (McWilliam, 1959). Homospecific pollen tubes grow normally and fertilize the eggs, whereas heterospecific pollen tubes lose their way. Such selective abilities for nucellus and its liquid secretion points to the fact that in some gymnosperms pollination drops are capable of recognition at a species level. There is some evidence to support the idea that some gymnosperms have either preadaptation or adaptations for mate selection of pollen (Willson and Burley, 1983). Recently, transcriptomic study of *C. sinensis* ovules during pollination drop secretion revealed a transcript that matched an S-locus lectin protein kinase, as well as four transcripts that matched a g-type lectin S-receptor-like serine /threonine kinase (Pirone-Davies et al., 2016). More work needs to be done on the molecular interactions of S-receptor-kinases in gymnosperms, if only because in some flowering plants, sporophytic self-incompatibility systems in Brassicaceae make much use of S-receptor kinase. They function as female determinants of male rejection.

## Perspectives

The purpose of this review was to summarize the many facets of sexual fluids in gymnosperms. Some aspects of these fluids are much better understood than others. To help future researchers in this area, we provide a number of points that we think are worth pursuing, if only to help shed further light on some of these highly successful adaptations of seed-plant reproduction.

### Nucellus Is the Major Filter of Reproduction in Gymnosperms

What are the essential molecular events within the nucellus with regards to pollination drop secretion? Gene expression studies, and proteomic profiles – the useful first steps to developing models of nucellus activity and regulation of prezygotic reproductive events – have yet to be undertaken. The nucellus is a workhorse of a tissue that not only is responsible for megasporogenesis, pollination drop secretion, pollen tube screening, but also part of megaspore wall formation and, later, ovular plug formation. Compared to the integument, which plays a much less active role, the nucellus is responsible for the bulk of ovule defenses and pollen-ovule interactions. How do nectar secretions in gymnosperms, i.e., pollination drop production, compare with the types of secretion by angiosperms? Can secretions be categorized according to known types, i.e., eccrine, merocrine (Vassilyev, 2010; Roy et al., 2017)? Do the processes that produce non-nectar differ from those that produce nectar? How extensively does the nucellus make use of enzymes that are widespread in angiosperm nectar regulation, such as invertases, e.g., CWIN4 (Heil, 2011), and sugar-transporters, such as SWEET9 (Roy et al., 2017)? Applying proteomics methods to pollination drops (Prior et al., 2013) has yielded many clues about pollination drop function. Such surveys should be expanded to include important clades, such as *Ginkgo*, cycads, and Podocarpaceae. The nucellus is not just involved in secretion, but also in resorption. How is resorption of pollination drops regulated? It seems that there are a number of possibilities, including slow responses, e.g., evaporation, and rapid responses. Do the latter involve ligand-gated ion channels?

### Molecular Clues in Nectar-Based Pollination Drops

In part because gymnosperm secretions have historically been considered to be abiotic or involved in gametophyte interaction only, it becomes important now to consider what other compounds are found in pollination drops, especially those drops that function as nectar. Analysis of lipids, terpenoids, and phenolics, all of which are known to occur in angiosperm nectar, have yet to be carried out on gymnosperm nectar. Measurements of phosphates (Ziegler, 1959) and volatile organic compounds that attract insects and geckos (Kato et al., 1995; Celedón-Neghme et al., 2016) need to be done. Analysis of compounds involved in animal pollination, which we now know extends back to mosses and ferns (Cronberg, 2012), should be initiated.

### Evolution of Time-Span Between Pollination and Fertilization

Compared to angiosperms, most gymnosperms invest more heavily in their prefertilization ovules. This adds developmental time (Leslie and Boyce, 2012) as a component of consideration compared to angiosperms in which the longest time from pollination to fertilization (i.e., vanilla orchid) is comparable to that of the fastest gymnosperms like *Ephedra* (Williams, 2012). As a result, the period between pollen capture and fertilization in a typical gymnosperm is relatively long. In more than a dozen genera it takes a year or more from pollen capture to gamete delivery (Willson and Burley, 1983; Williams, 2012). So how is it that some Gnetales with a PCM α-type pollination drop can trigger germination within a day of pollen capture (El-Ghazaly et al., 1998)? What are, in fact, the molecular controls of germination? What are the advantages and disadvantages of these various pollination to fertilization periods?

### Ancient Origins of Gymnosperm Sexual Fluids and Nectar

From what we now know about ovule evolution, we can pose some new questions. Did the earliest Paleozoic seed plants such as hydraspermans have one or two sexual fluids? Did the earliest plants in the Devonian release sperm immediately upon capture of their prepollen, or was prepollen held for a time before release of swimming sperm? Was the fertilization fluid associated with a reproductive system in which microgametophytes reached maturity long after pollination before fertilizing eggs in later developed megagametophytes, as is seen in modern cycads and *Ginkgo*, or was the fertilization fluid part of single multi-purpose fluid in which the sexual fluid would have functioned as a PCM and as a fertilization fluid? If it was the latter, then it would suggest that the earliest ovules produced a single fluid having the functions of prepollen capture, delivery, germination, and ovule defense, as well as the function of a swimming sperm medium.

## Conclusion

The two general types of sexual fluids in gymnosperms are pollination drops and fertilization fluids during fertilization. Both occur in ovules. The fertilization fluid originates from gametophytic tissues. We know less about these particular fluids in modern seed-plants, because we still await chemical analysis of their composition. We know much more about pollination drops. The plesiomorphic pollination syndrome of modern gymnosperms may share features with those of the earliest gymnosperms (i.e., PCM α). Pollination drops represent a significant investment in a fluid by the sporophytic tissues of the ovule. Drops have numerous functions in relatively complex PCMs: they ensure pollen capture, transport, germination and selection, ovule defense, and in some species, nectar reward for pollinators. The ability to present the drop as a nectar is found in three of the four major extant clades of gymnosperms, including the two most ancient ones (Ginkgoales, Cycadales). Nectar production may well have also been present in the distant past. We are beginning to understand elements of drop physiology, such as secretion and retraction. As we increase our knowledge of the regulation of secretion we will also begin to broaden our appreciation of nectar secretion by ovules as a unique and important contribution of gymnosperms to the evolution of seed plants.

## Author Contributions

All authors listed have made a substantial, direct and intellectual contribution to the work, and approved it for publication.

## Conflict of Interest Statement

The authors declare that the research was conducted in the absence of any commercial or financial relationships that could be construed as a potential conflict of interest.
